# Coupling *S*-adenosylmethionine–dependent methylation to growth: Design and uses

**DOI:** 10.1371/journal.pbio.2007050

**Published:** 2019-03-11

**Authors:** Hao Luo, Anne Sofie L. Hansen, Lei Yang, Konstantin Schneider, Mette Kristensen, Ulla Christensen, Hanne B. Christensen, Bin Du, Emre Özdemir, Adam M. Feist, Jay D. Keasling, Michael K. Jensen, Markus J. Herrgård, Bernhard O. Palsson

**Affiliations:** 1 Novo Nordisk Foundation Center for Biosustainability, Technical University of Denmark, Kongens Lyngby, Denmark; 2 Department of Bioengineering, University of California, San Diego, La Jolla, California, United States of America; 3 Joint BioEnergy Institute, Emeryville, California, United States of America; 4 Center for Synthetic Biochemistry, Institute for Synthetic Biology, Shenzhen Institutes of Advanced Technologies, Shenzhen, China; 5 Biological Systems & Engineering Division, Lawrence Berkeley National Laboratory, Berkeley, California, United States of America; 6 Department of Chemical and Biomolecular Engineering and Department of Bioengineering, University of California, Berkeley, California, United States of America; 7 Department of Pediatrics, University of California, San Diego, La Jolla, California, United States of America; Stanford University, United States of America

## Abstract

We present a selection design that couples *S*-adenosylmethionine–dependent methylation to growth. We demonstrate its use in improving the enzyme activities of not only N-type and O-type methyltransferases by 2-fold but also an acetyltransferase of another enzyme category when linked to a methylation pathway in *Escherichia coli* using adaptive laboratory evolution. We also demonstrate its application for drug discovery using a catechol O-methyltransferase and its inhibitors entacapone and tolcapone. Implementation of this design in *Saccharomyces cerevisiae* is also demonstrated.

## Author summary

Many important biological processes require methylation, e.g., DNA methylation and synthesis of flavoring compounds, neurotransmitters, and antibiotics. Most methylation reactions in cells are catalyzed by *S*-adenosylmethionine (SAM)–dependent methyltransferases (Mtases) using SAM as a methyl donor. Thus, SAM-dependent Mtases have become an important enzyme category of biotechnological interests and as healthcare targets. However, functional implementation and engineering of SAM-dependent Mtases remains difficult and is neither cost effective nor high throughput. Here, we are able to address these challenges by establishing a synthetic biology approach, which links Mtase activity to cell growth such that higher Mtase activity ultimately leads to faster cell growth. We show that better-performing variants of the examined Mtases can be readily obtained by growth selection after repetitive cell passages. We also demonstrate the usefulness of our approach for discovery of Mtase-specific drug candidates. We further show our approach is not only applicable in bacteria, exemplified by *Escherichia coli*, but also in eurkaryotic organisms such as budding yeast *Saccharomyces cerevisiae*.

## Introduction

Methylation is the transfer of a methyl group from one molecule to another. Its importance in gaining bioactivity and acquiring bioavailability of drugs has been recognized by chemists for some time [1]. Chemical methylation ordinarily utilizes noxious reagents and generates toxic waste and often lacks regioselectivity [2]. In contrast, enzymatic methylation is specific, environmentally friendly, and safer to work with.

Most methylation in cells takes place by *S*-adenosylmethionine–dependent methyltransferases (SAM-dependent Mtases), using SAM as the methyl donor. SAM-dependent methylation is involved in many important biological processes, including epigenetics and synthesis of a wide range of secondary metabolites (e.g., flavonoids, neurotransmitters, antibiotics). In fact, SAM is one of the most commonly used cofactors in cellular metabolism, second only to ATP [3]. SAM-dependent Mtases have become an important enzyme category, used either as biocatalysts, as part of fermentative production pathways in biotechnical and chemical industries [2–4], or as drug targets in the pharmaceutical industry [5,6].

**Fig 1:**
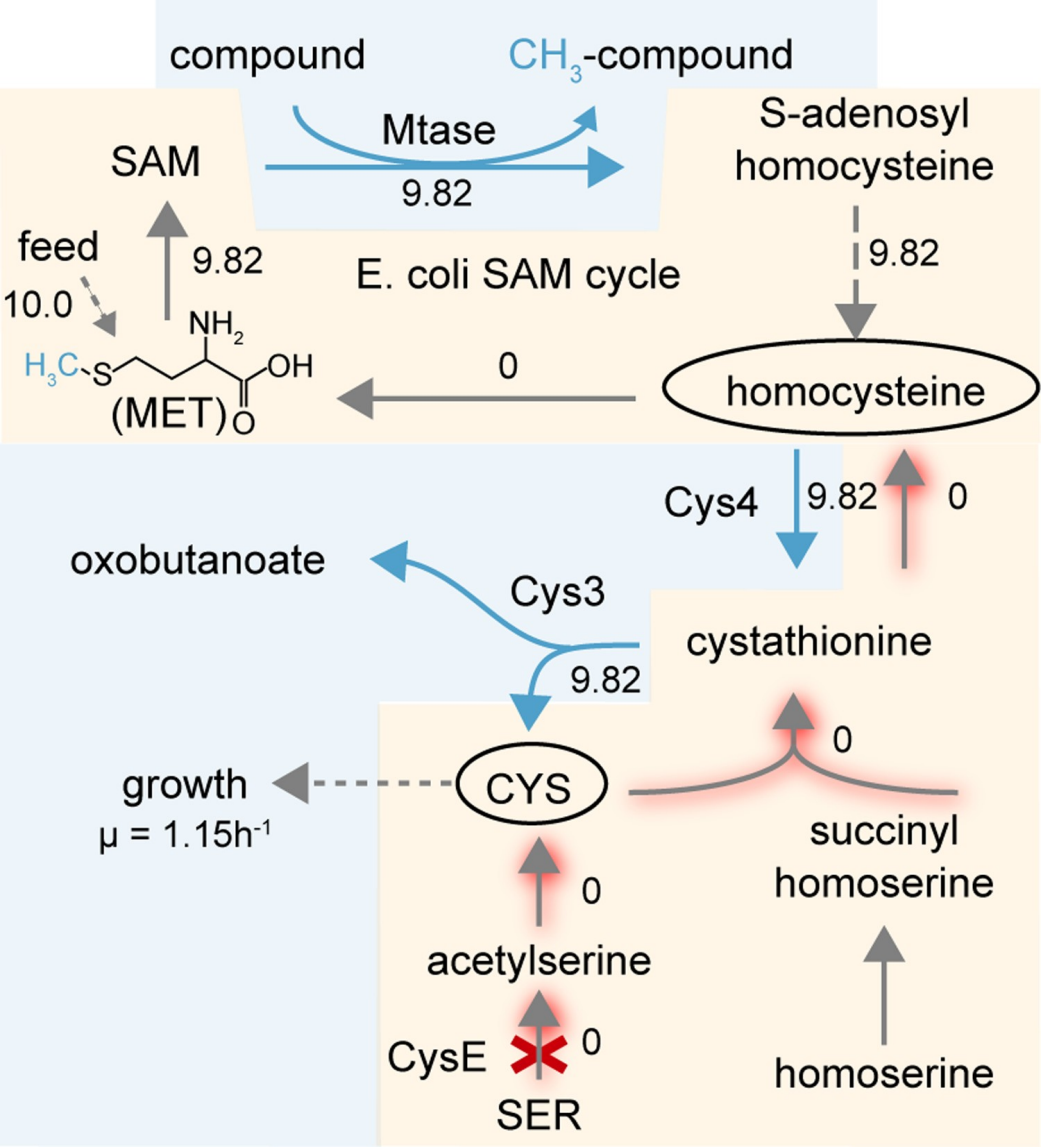
Illustration of growth-coupled methyltransferase selection system design in *E*. *coli*. Native reactions are colored in gray, and heterologous reactions are colored in cyan. Dashed lines represent multiple enzymatic reactions. Red shaded lines indicate an inactive pathway after gene deletion (red cross). Metabolically related reactions are boxed together. Numbers shown are simulated metabolic fluxes as mmol gram per dry weight per hour. μ indicates the growth rate. CYS, cysteine; *cysE*, serine acetyltransferase; Cys3, cystathionine-γ-lyase; Cys4, cystathionine-β-synthase; MET, methionine; Mtase, methyltransferase; SAM, *S*-adenosylmethionine; SER, serine.

Implementing and engineering functional SAM-dependent Mtases is difficult since all existing assays lack robustness, are not cost effective, and are not generalizable to all types of Mtases or high throughput. Consequently, study and engineering of Mtases or building efficient methylation-dependent pathways is hard to achieve. We designed an in vivo synthetic selection system by coupling SAM-dependent methylation to growth via a homocysteine intermediate of the SAM cycle. This selection system design was first implemented in *E*. *coli* by deleting serine acetyltransferase (*cysE*) to prevent endogenous homocysteine and cysteine synthesis. We diverged homocysteine of the SAM cycle to cysteine using heterologously expressed yeast cystathionine-β-synthase (Cys4) and cystathionine-γ-lyase (Cys3) (Figs 1 and S1). Effectively, this design couples methylation to the conversion of exogenous methionine to the biosynthesis of cysteine, a required amino acid for growth. This growth-coupled design was computationally validated using a genome-scale metabolic model (Fig 1).

## Results

**Fig 2:**
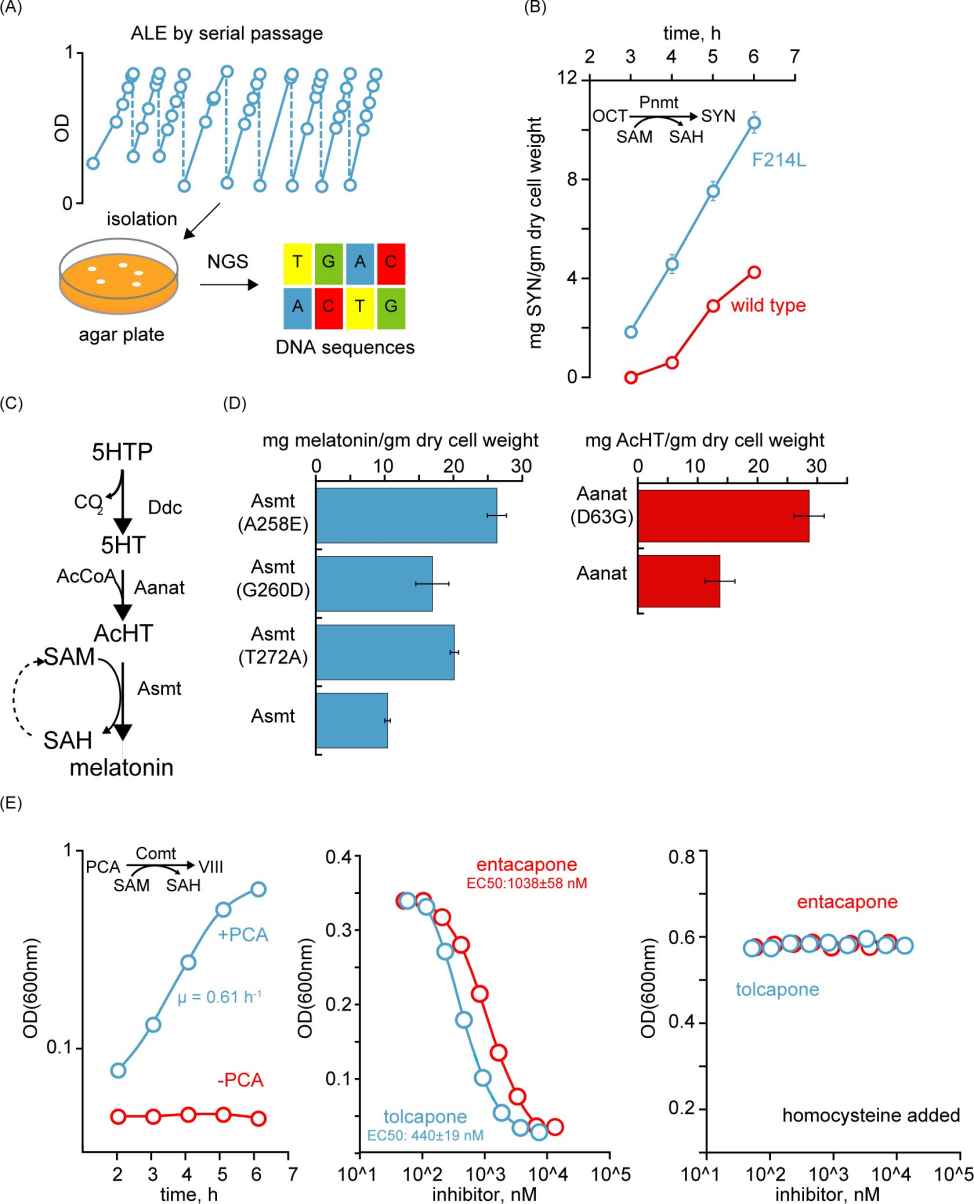
Uses of the Mtase selection system. (A) ALE-driven workflow. (B) In vivo enzymatic comparison of wild-type Pnmt and variant shown in time course. *n* = 4, and error bars indicate SD. (C) The melatonin pathway. (D) In vivo enzymatic activity of Asmt and Aanat variants after 6 h cell growth. See S2 Fig for details. *n* = 3, and error bars indicate SD. (E) Comt-dependent growth shown using an evolved isolate bearing RpoC (A328P). μ indicates the growth rate. Inhibitor titration curves of the same strain in the presence or absence of homocysteine. *n* = 4, and error bars are SD. Underlying data can be found in S1 Data. Aanat, aralkylamine N-acetyltransferase; AcHT, acetylserotonin; AcCoA, acetyl-CoA; ALE, adaptive laboratory evolution; Asmt, acetylserotonine O-methyltransferase; Comt, catechol O-methyltransferase; Ddc, aromatic-amino-acid decarboxylase; Mtase, methyltransferase; NGS, next-generation sequencing; OCT, octopamine; OD, optical density; PCA, protocatechuic acid; Pnmt, phenylethanolamine N-methyltransferase; RpoC, RNA polymerase subunit beta; SAH, *S*-adenosylhomocysteine; SAM, *S*-adenosylmethionine; SD, standard deviation; SYN, synephrine; VIII, vanillic acid; 5HT, serotonin; 5HTP, 5-hydroxytryptophan.

We demonstrate the use of our system to improve enzyme properties. Using adaptive laboratory evolution (ALE) with growth selection, we first achieved directed evolution of phenylethanolamine N-methyltransferase (Pnmt) using a non-natural substrate, octopamine (OCT). In recent years, ALE has emerged as a productive approach to address a wide range of biological questions [7–9]. We implemented an ALE-driven workflow to demonstrate our selection system because of the operational simplicity of ALE (serial passages), its ultra-high–throughput screening capability (over 10 million cells per passage), its ability to engage phenotype-driven in vivo evolution, and the affordability of DNA resequencing (Fig 2A). Following ALE, isolated strains were characterized and were subjected to full DNA resequencing. Mutations in *E*. *coli cfa*, involved in phospholipid synthesis, were identified in all non-growth–coupled isolates. The *cfa* gene encodes for a SAM-dependent Mtase, suggesting its role as a competing Mtase during ALE. On the other hand, a Pnmt (F214L) mutation was present in growth-coupled isolates, and a cell-based characterization showed that it led to approximately 2-fold activity improvement on synephrine (SYN) synthesis (Fig 2B).

Most often, Mtase substrates may not be readily available in large quantities or even membrane permeable in order to perform directed enzyme evolution in vivo, and it may then only be feasible to engage an active metabolic pathway. We thus applied our selection system to evolve a methylation-dependent pathway. The chosen candidate pathway was a de novo three-step melatonin biosynthesis pathway from 5-hydroxytryptophan (5HTP). It consisted of three enzymatic steps: decarboxylation (aromatic-amino-acid decarboxylase [Ddc]), acetylation (aralkylamine N-acetyltransferase [Aanat]), and methylation (acetylserotonine O-methyltransferase [Asmt]) (Fig 2C). By using ALE, Asmt was evolved in vivo, and three sequence variants (A258E, G260D, and T272A) were discovered (Fig 2D). All variants showed improved turnover compared to wild-type Asmt under physiological conditions, with the highest improvement observed in A258E (approximately 2.5-fold). It was additionally discovered that high levels of *ddc* expression from a plasmid caused genetic instability, and mutations in *cfa* could be seen in non-melatonin–producing cells, affirming its role as an unwanted sink for SAM in *E*. *coli*. Upon incorporating Asmt (A258E), a single copy of *ddc*, and *cfa* deletion in the background strain, Aanat was further evolved in the next ALE, and the D63G mutation was identified, leading to approximately 2-fold activity improvement (Fig 2D). These results demonstrated the usefulness of this growth selection system for directed evolution of enzymes or metabolic pathways when linked to a methylation reaction.

We next demonstrate the use of our system for drug discovery. SAM-dependent Mtases participate in many important cellular functions and are targeted by a number of drug development programs (such as DNA or histone Mtase inhibitors) [6]. We applied our selection system on catechol O-methyltransferase (Comt), a known drug target for treating Parkinson's disease [5]. Cells bearing human Comt were evolved to grow at high rates using ALE (Fig 2E). All isolates were growth-coupled to Comt activity. Resequencing results showed the *comt* gene did not acquire any mutations, while many isolates accumulated mutations on RpoC (such as A328P, E1146A, or E1146G), a subunit of *E*. *coli* RNA polymerase, suggesting a host factor effect. The suitability of using evolved cells to screen Comt inhibitors by growth was evaluated next by determining Z-factor in a 96-well format [10]. The Z-prime value was calculated to be between 0.87 to 0.97 when cells were grown for 3 h or more, indicating a high-throughput-screening (HTS)–compatible assay with large separation (Fig 2E and S1 Table). We then tested one evolved isolate with two known Comt inhibitors: entacapone and tolcapone, respectively. Both drugs reduced Comt-dependent cell growth at concentrations as low as 200 nM, with a slightly higher potency observed for tolcapone (Fig 2E). Both inhibitors were highly specific to Comt and showed no observable adverse effects on other cellular proteins (such as heterologous Cys3 and Cys4 or the essential *E*. *coli* proteins) when homocysteine was additionally supplemented, implying a general suitability of our selection system for in vivo Comt inhibitor screening (Fig 2E).

**Fig 3:**
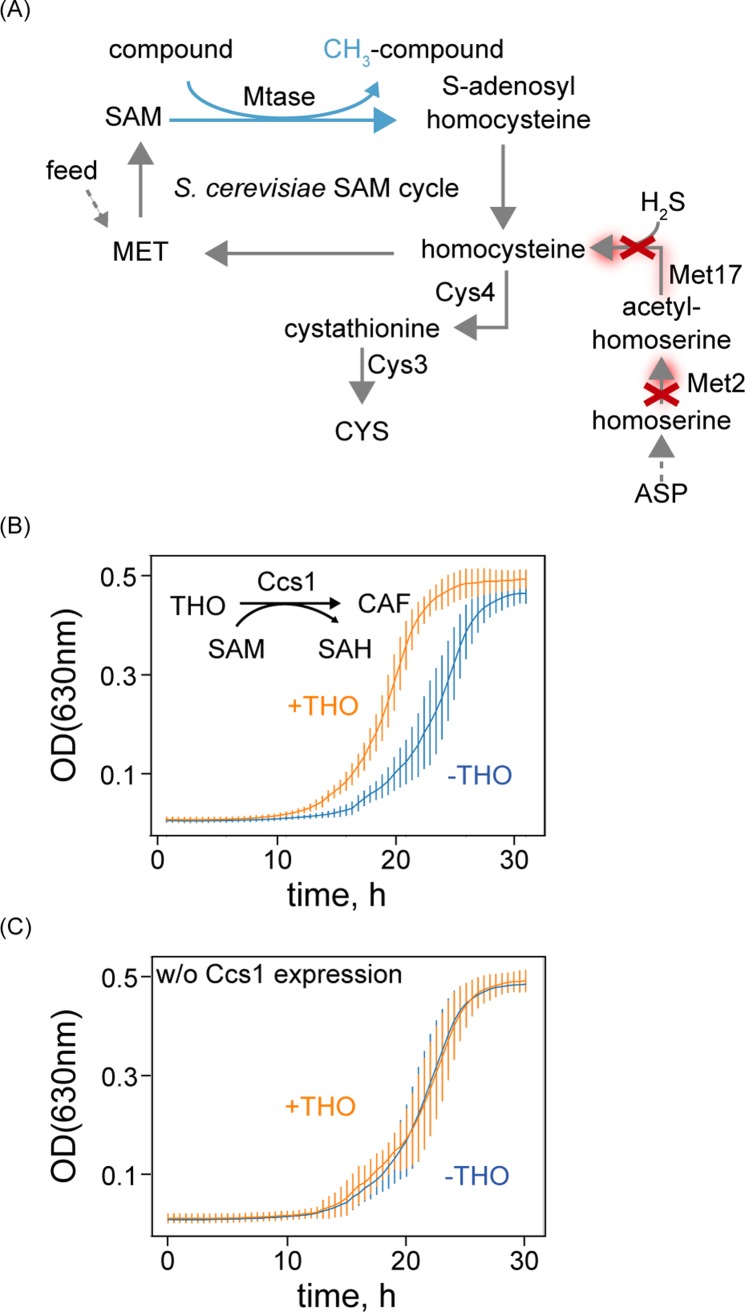
Demonstration of Mtase selection design in *S*. *cerevisiae*. (A) Illustration of growth-coupled Mtase selection design in *S*. *cerevisiae*. Native reactions are colored in gray, and heterologous reactions are colored in cyan. Dashed lines represent multiple enzymatic reactions. Red crosses indicate gene deletion. Red shaded lines indicate inactive reactions after gene deletion (red cross). Cofactors, byproducts, and co-substrates are omitted. Growth of *S*. *cerevisiae* expressing heterologous Ccs1 (B) and empty plasmid control cells (C) in the presence (orange) and absence (blue) of theobromine with methionine. Error bar indicates SD. Underlying data can be found in S1 Data. ASP, aspartic acid; CAF, caffeine; *CCS1*, caffeine synthase I; CYS, cysteine; Cys3, cystathionine-γ-lyase; Cys4, cystathionine-β-synthase; MET, methionine; Mtase, methyltransferase; OD, optical density; SAH, *S*-adenosylhomocysteine; SAM, *S*-adenosylmethionine; THO, theobromine; w/o, without.

Lastly, we implemented our design in budding yeast *S*. *cerevisiae*. *S*. *cerevisiae* is an industrially important production host with growing interest for biobased production of value-added methylated products [11]. It is also a well-studied eukaryotic model organism expressing diverse cellular Mtases [12]. In contrast to *E*. *coli*, yeast is capable of synthesizing cysteine through reverse transsulfuration from homocysteine because of the natural appearance of the *CYS3* and *CYS4* genes (Fig 3A) [13]. Therefore, blockage of homocysteine biosynthesis from aspartate is required to enable the selection, and this was achieved by deleting the genes encoding homoserine O-acetyltransferase (*MET2*) and O-acetylhomoserine sulfhydrylase (*MET17*). Additional gene deletion of phosphatidylethanolamine methyltransferase (*CHO2*) and phospholipid methyltransferase (*OPI3*) required for phosphatidylcholine biosynthesis was performed to remove potential competing native Mtases for SAM [14]. In this quadruple knock-out strain, the heterologous caffeine synthase I gene (*CCS1*), encoding an N-Mtase from *Coffea arabica* acting on theobromine to synthesize caffeine, was introduced. The presence of Ccs1 conferred growth advantage when exogenous theobromine was supplemented compared to nonsupplemented cells, affirming the applicability of the design in yeast (Fig 3B). Control cells without Ccs1 expression showed similar growth regardless of theobromine supplementation (Fig 3C). Acknowledging the large number of native Mtases in yeast [12], this phenotype might be the result of the activity of remaining native Mtases for homocysteine synthesis required for growth.

## Discussion

In summary, we have designed and validated a methylation-dependent growth selection system for Mtases. Not only did this selection system lead to the discovery of causal mutations that improve enzyme properties of heterologous Mtases and acetyltransferase, but it also demonstrated usefulness in drug discovery and the identification of critical host factors. Further applications of this technology can be used to acquire in-depth understanding of Mtases (such as their evolutionary path and governing factors for substrate specificity) or to engineer pathways or host cells of metabolic engineering interests. We have implemented our design in *E*. *coli* and *S*. *cerevisiae*; however, the conceptual design of this selection is transferable to any organism with an active SAM cycle. Implementing this selection system in higher eukaryotic cells (such as mammalian cells) is particularly valuable for drug development since permeability and stability of promising drug candidates can be determined at early stages. Overall, the described selection is robust, generalizable, compatible with HTS, and widely applicable, and it is likely to become a useful and valuable tool for the chemical biology and metabolic engineering communities.

## Materials and methods

### Microbial strains, media, and growth conditions

The *E*. *coli* BW25113 strain and derivatives were used throughout this study. Genome engineering of *E*. *coli* was facilitated by λ-red recombination, P1 transduction, and/or site-specific Tn7 transposon [15,16]. *S*. *cerevisiae* CEN.PK 102-5B (*MAT*a) was used as background yeast strain for the engineered yeast strains. Genome engineering of *S*. *cerevisiae* was facilitated by CRISPR/Cas9 [17,18], and transformations were performed with the lithium acetate/single-stranded carrier DNA/PEG method [19]. A list of strains used is shown in S2 Table. Unless stated otherwise, all *E*. *coli* strains were maintained at 37°C in LB (Lennox) (Sigma Aldrich, St. Louis, MO, USA), 2xYT, or M9 media containing M9 minimal salts (BD Difco, BD, Franklin Lakes, NJ, USA), 2 mM MgSO_4_, 100 μM CaCl_2_, 500-fold diluted trace minerals (10 g/L FeCl_3_·6H_2_O, 2 g/L ZnSO_4_·7H_2_O, 0.4 g/L CuCl_2_·2H_2_O, 1 g/L MnSO_4_·H_2_O, 0.6 g/L CoCl_2_·6H_2_O, and 1.6 mM EDTA [pH 8.0]), 1× ATCC Vitamin Supplement (ATCC MD-VS), and 0.2% glucose (w/v). Unless stated otherwise, ampicillin and spectinomycin were used at 50 mg/L; chloramphenicol and kanamycin were used at 25 mg/L. Yeast strains were cultured on either rich yeast extract-peptone dextrose (YPD), yeast synthetic drop-out media (lacking the proper amino acids for selection) (Sigma Aldrich), or Delft medium [20] with 2% glucose. Delft medium was sterilized by filtration. All *Δcho2*- and *Δopi3*-derived strains were maintained on media supplemented with 1 mM choline chloride (Sigma Aldrich), except for YPD medium.

### Plasmids

Plasmid DNA assemblies were performed by Gibson Assembly, USER cloning, or the EasyCloning method [20–22]. *E*. *coli* TOP10 (Invitrogen, Carlsbad, CA, USA) and DH5α were used for plasmid propagations. For propagation of yeast plasmids, 100 mg/L ampicillin was used. A list of plasmids used is summarized in S3 Table.

### ALE

All ALE experiments were performed at 37°C using M9 supplemented with 50 mg/L methionine and substrates, unless stated otherwise. Cell passages were performed automatically according to previously described [23], and six lineages of the initial strain were usually maintained. OCT and PCA at 50 mg/L were supplemented for Pnmt and Comt evolution, respectively. The melatonin pathway was evolved in the presence of 100 mg/L of methionine and 100 mg/L of 5HTP. Antibiotics were not supplied during ALE.

### Strain construction for methylation-dependent selection

The *E*. *coli* ECAH2 strain (ΔcysE), derived from JW3582 of the Keio Collection [24], was used as the host strain to test methylation-dependent growth in the presence of pHM11. The pHM11 plasmid harbored *cys3* and *cys4* from *S*. *cerevisiae* S288C. Homocysteine and Cys3/Cys4-dependent cysteine synthesis was demonstrated by transforming ECAH2 with pHM11. The transformed strain, ECAH3, was allowed to grow on an M9 plate containing 50 mg/L homocysteine at 37°C for 24 h to demonstrate homocysteine-dependent growth. The ECAH6 and ECAH7 strains were used for Pnmt and Comt evolution using ALE. All ΔcysE-derived strains were maintained on LB plates supplemented with 25 mg/L cysteine.

The *E*. *coli* HMP236 strain was initially used to evolve the melatonin pathway from 5HTP (S2 Table). It contained two plasmids, pHM11 and pHM12. Its parent strain was HMP221 with the following genome modifications: FolE (T198I), YnbB (V197A), ΔtnaA, ΔcysE, ΔmetE, and ΔmetH. Deletion of *tnaA* was to prevent 5HTP degradation. Deletion of *metE* and *metH* was to prevent a reverse methionine-to-homocysteine synthesis via methionine synthase encoded by both genes, but it was later determined not to be required.

The *E*. *coli* HMP579 was used for the subsequent melatonin pathway evolution. It carried two plasmids, pHM70 and pHM79. The pHM70 plasmid was modified from pHM11 with an insertion of Asmt (A258E). The second plasmid, pHM79, was not required in this study because of 5HTP feeding. Its parent strain was HMP553, carrying a single chromosomal copy of *ddc* and *aanat*. The *ddc* and *aanat* genes were introduced to the attTn7 site using pGRG25.

The *S*. *cerevisiae* SCAH124 strain, derived from *S*. *cerevisiae* CEN.PK102-5B (*MAT*a), was constructed by sequential CRISPR/Cas9-facilitated full ORF markerfree knock-out of *MET17*, *CHO2*, *OPI3*, and *MET2* mediated by homology-directed recombination using guide RNA (gRNA)-expressing plasmids PL_01_A2, PL_01_A3, PL_01_C8, and PL_01_E1 and two linear DNA fragments homologous to the flanking regions up- and downstream of the targeted ORF, as well as PL_01_A9 with *CAS9*. The following gRNA sequences were used for Cas9-targeting of indicated ORF and identified with the webservice CRISPy adapted for the *S*. *cerevisiae* CEN.PK genome sequence [25]: *MET17* (5′-GATACTGTTCAACTACACGC-3′), *CHO2* (5′-ACCACCTGTAACCCACGATA-3′), *OPI3* (5′- GCAGAAACAACCAGCCCCGC-3′) and *MET2* (5′- GTAATTTGTCATGCCTTGAC-3′). SCAH134 and SCAH138, derived from SCAH124 and harboring the Mtase gene *CCS1* (PL_01_D2) and an empty vector (pRS415U), respectively, were used for demonstration of selection design in *S*. *cerevisiae*.

### DNA resequencing and data analysis

Total DNA was extracted using a PureLink Genomic DNA Kit (Invitrogen) and was processed either commercially by Beckman Coulter Genomics (Danvers, MA, USA) or in house. When prepared in house, DNA libraries were prepared using a Kapa Hyper Prep Library Prep Kit (Roche Molecular Systems, Pleasanton, CA, USA). DNA samples were sequenced using Illumina MiSeq or NextSeq.

Trimmomatic tool (v0.32-v0.35) was used for quality trimming of raw sequencing data with "CROP:145 HEADCROP:15 SLIDINGWINDOW:4:15 MINLEN:30" parameters [26]. Breseq (v0.27.1) was employed for variant calling on processed sequencing data with "-j 4 -b 20" parameters [27]. *E*. *coli* BW25113 genome sequence with NCBI accession CP009273 was used as reference along with other relevant parts and plasmids.

### Compound analysis

All compounds were purchased from Sigma Aldrich. PCA and its methylated product vanillic acid (VIII) were quantified using a Dionex 3000 HPLC system (Dionex, Sunnyvale, CA, USA) equipped with a Cortecs UPLC T3 column from Waters (Milford, MA, USA) and a guard column from Phenomenex (Torrance, CA, USA). The column temperature was set to 30°C, and the mobile phase consisted of a 0.1% formic acid and acetonitrile. Runtime was 11 min, including 4.2 min separation without acetonitrile, 1 min washing with 75% acetonitrile, and an additional 4.5 min run without acetonitrile. The flow rate was constant at 0.3 ml/min, and the injection volume was 1 μl. Both compounds were detected by UV at wavelengths 210, 240, and 300 nm as well as a 3D UV scan. HPLC data were processed using Chromeleon 7.1.3 software (Thermo Fisher Scientific, Waltham, MA, USA), and compound concentrations were calculated using calibration curves.

5HTP, serotonin (HT), acetylserotonin (AcHT), and melatonin were quantified using a Dionex 3000 HPLC system equipped with a Zorbax Eclipse Plus C18 column (Agilent Technologies, Santa Clara, CA, USA) and a guard column from Phenomenex. To achieve separation, the column was heated to 30°C, and the mobile phase consisted of a 0.05% acetate and a variable amount of acetonitrile. Runtime was 12 min, including 10 min of separation, whereas acetonitrile was reduced from 95% to 38.7% in 9.4 min. After holding 0.6 min, acetonitrile concentration was returned to 95% in 1 min and was held till the end of the run. The flow rate was set to 1 ml/min, and the injection volume was 1 μl. Elution of the compounds was detected by UV at wavelengths 210 nm, 240 nm, 280 nm, and 300 nm as well as a 3D UV scan. HPLC data were processed using Chromeleon 7.1.3 software (Thermo Fisher Scientific), and compound concentrations were calculated using calibration curves.

OCT was quantified using a Dionex 3000 HPLC system equipped with a Cortecs UPLC T3 column from Waters and a guard column from Phenomenex. The column temperature was set to 30°C, and the mobile phase consisted of 0.1% formic acid and acetonitrile. Runtime was 9 min, including 2.5 min separation without acetonitrile, 0.5 min washing with 70% acetonitrile, and an additional 5.5-min run without acetonitrile. The flow rate was constant at 0.3 ml/min, and the injection volume was 1 μl. OCT was detected by UV at wavelengths 210, 240, and 300 nm as well as a 3D UV scan. SYN was detected by LC-MS (Fusion, Thermo Fisher Scientific) in the positive full-scan mode using the same separation profile as OCT quantification. SYN was detected as [M + H]^+^ m/z 168.10191 with a mass accuracy of 2.2 ppm. Data were processed using Chromeleon 7.1.3 and X-calibur 4.1 from Thermo Fisher Scientific, and compound concentrations were calculated using calibration curves.

### Growth measurements

Growth of *E*. *coli* was measured using a Duetz 96-well low well system (Enzyscreen, Heemstede, The Netherlands) coupled to a humidified Innova 44 shaker (5 cm orbit) (New Brunswick Scientific, Edison, NJ, USA) at 37°C and 300 rpm. Seed cells were grown in 400 μl LB in the presence of appropriate antibiotics for 4–5 h in 96-well deep well plates. When transferred to M9, 10 μl of cells were added to 400 μl of M9 with 25 mg/L cysteine and antibiotics. After overnight growth, cells were added to 150 μl of fresh M9 with 50 mg/L of methionine and 50 mg/L methylation substrates to approximately 4% in a 96-well low well plate. Changes in optical density at 600 nm (OD600) were recorded using a SynergyMx microplate reader (BioTek Instruments, Winooski, VT, USA). Growth rates were calculated from an average of four independent biological replicates using KaleidaGraph 4.1.3.

Six biological replicates of *S*. *cerevisiae* SCAH134 and SCAH138 were inoculated from seed cultures to similar ODs for sulfur amino acid starvation in Delft medium supplemented with histidine, uracil, and choline chloride and incubated for approximately 24 h. The seed cultures were grown in yeast synthetic complete medium without leucine, supplemented with choline chloride, for approximately 25 h. Both replicate seed cultures and starvation cultures were cultured in a total volume of 500 μl in 96-deep well plates at 30°C/300 rpm. Upon completion of starvation, cells were transferred to Delft medium supplemented with histidine, uracil, choline chloride, 1 mM L-methionine, and with/without 1 mM theobromine (Sigma Aldrich) to similar ODs and to a final volume of 150 μl in a flat-bottomed microtiterplate. Two technical replicates were inoculated for each of the six biological replicates. Growth was recorded in a microtiterplate reader (ELx808 Absorbance Reader, BioTek Instruments) with continuous shaking at strong setting, at 30°C, and with recording of absorbance at 630 nm every 30 min. The cultures had incubated in the plate reader 30 min prior to the first reading at time 0 h. Growth curves were plotted using mean absorbance of the replicates for 30 h. Blank media values were not subtracted.

### Growth inhibition assay using entacapone and tolcapone

HL1818 was used to test the inhibition effect of entacapone and tolcapone. Cells were prepared for growth measurement as described above. The test growth medium was M9 with 50 mg/L PCA, 50 mg/L methionine, 1% DMSO, and various amount of inhibitors. The stock concentration of entacapone and tolcapone was 20 g/L dissolved in DMSO. To the positive controls, 50 mg/L homocysteine was additionally included so that effects of the drugs on *E*. *coli* cells (such as those other than Comt) could be determined. A drug concentration response curve was plotted using average OD600 after 6 h growth from three independent biological replicates. The EC50 values were calculated using OriginPro 2018b (version b9.5.5.409).

### Characterizations of Pnmt activity in vivo

Measurements of SYN production were performed using a Duetz 96-well deep well system (Enzyscreen) coupled to an Innova 44 shaker (5 cm orbit) (New Brunswick Scientific) at 37°C and 300 rpm. HL1815 and HL1816 were used to measure SYN production (S1 Table). Seed cells were grown in LB in the presence of chloramphenicol for 4–5 h and thereafter grew in M9 overnight. Fresh M9 containing 200 mg/L OCT was inoculated with seed culture to 4%. These cells were transferred to a 96-well deep well plate, and each well contained 400 μl. 200 μl samples were withdrawn periodically for exometabolites analysis, while the remaining 200 μl cells were used to determine OD values using a SynergyMx microplate reader (BioTek). In vivo enzyme activity was averaged from four independent biological measurements normalized to dried cell weight. The conversion factor from OD to dried cell weight is 1 (i.e., 1 OD = 1 g/L) for our setup. It was observed that Pnmt activity was biomass dependent.

### Characterizations of Asmt and Aanat activity in vivo

A Duetz 24-well deep well system (Enzyscreen) coupled to an Innova 44 shaker (5 cm orbit) (New Brunswick Scientific) was used. Physiological Asmt activity was determined by growing HMP231, HMP416, HMP416, HMP417, and HMP418 in 2 ml M9 supplemented with 100 mg/L AcHT at 37°C with shaking at 300 rpm. Samples were withdrawn periodically for exometabolites analysis using HPLC and OD measurements. Physiological Aanat activity was measured using HMP850 and HMP851 in the presence of 100 mg/L HT. In vivo enzyme activity was averaged from three independent biological measurements normalized to dried cell weight. It was observed that Asmt and Aanat activity was biomass independent.

### Validation of selection systems using an *E*. *coli* genome-scale metabolic model

Validation of the selection system design was performed using the most recent *E*. *coli* genome-scale metabolic model [28], which computes the flux states of the entire metabolic network. Following established procedures [29], the *cysE* gene was “knocked out” in silico by setting the upper and lower bounds of the metabolic reaction it catalyzes to 0. Metabolic reactions catalyzed by Cys3 and Cys4 were inserted into the metabolic model. Additionally, a methylation-dependent reaction was inserted into the model (Pnmt, Comt, or Asmt). The metabolic model was then solved for its flux state using linear programming by setting cell growth as the objective. All model simulations were performed using the python package COBRApy 0.7.0 in Python 2.7 [30].

## Supporting information

S1 Fig Homocysteine-dependent growth with heterologous expression of Cys3 and Cys4. Cys3, cystathionine-γ-lyase; Cys4, cystathionine-β-synthase.
(TIF)

S2 Fig Time-course measurements of Asmt and Aanat activity in vivo. Underlying data can be found in S1 Data. Aanat, aralkylamine N-acetyltransferase; Asmt, acetylserotonine O-methyltransferase.
(TIF)

S1 Table Calculated Z-prime values for evolved Comt isolates at various time of growth. Comt, catechol O-methyltransferase.
(DOCX)

S2 Table List of strains used in this study. (DOCX)

S3 Table List of plasmids used in this study. (DOCX)

S1 Data Data underlying Figs 2 and 3 and S2. (XLSX)

## Abbreviations

Aanat: aralkylamine N-acetyltransferase
AcCoA: acetyl-CoA
AcHT: acetylserotonin
ALE: adaptive laboratory evolution
Asmt: acetylserotonine O-methyltransferase
ASP: aspartic acid
CCS1: caffeine synthase I
CHO2: phosphatidylethanolamine methyltransferase
Comt: catechol O-methyltransferase
CYS: cysteine
cysE: serine acetyltransferase
Cys3: cystathionine-γ-lyase
Cys4: cystathionine-β-synthase
Ddc: aromatic-amino-acid decarboxylase
gRNA: guide RNA
HT: serotonin
HTS: high-throughput screening
MET: methionine
MET2: homoserine O-acetyltransferase
MET17: O-acetylhomoserine sulfhydrylase
Mtase: methyltransferase
NGS: next-generation sequencing
OCT: octopamine
OD: optical density
OPI3: phospholipid methyltransferase
PCA: protocatechuic acid
Pnmt: phenylethanolamine N-methyltransferase
RpoC: RNA polymerase subunit beta
SAH: *S*-adenosylhomocysteine
SAM: *S*-adenosylmethionine
SD: standard deviation
SER: serine
SYN: synephrine
VIII: vanillic acid
YPD: yeast extract-peptone dextrose
5HTP: 5-hydroxytryptophan
