# Supplementary figures and images for: Coupling S-adenosylmethionine–dependent methylation to growth: Design and uses
Source: PLoS Biol. 2019 Mar 11;17(3):e2007050. doi: 10.1371/journal.pbio.2007050 (PMC6411097; doi:10.1371/journal.pbio.2007050)

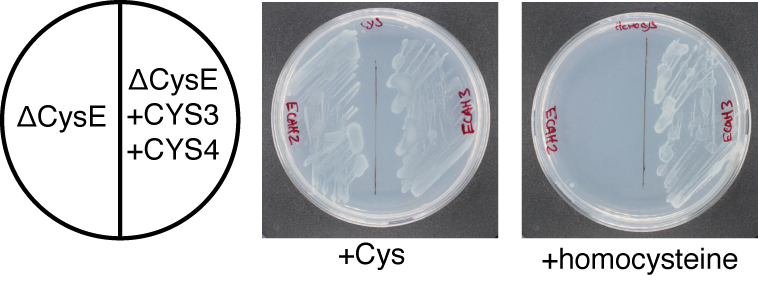

Supplement: S1 Fig — Cys3, cystathionine-γ-lyase; Cys4, cystathionine-β-synthase. (TIF) [file pbio.2007050.s001.tif]

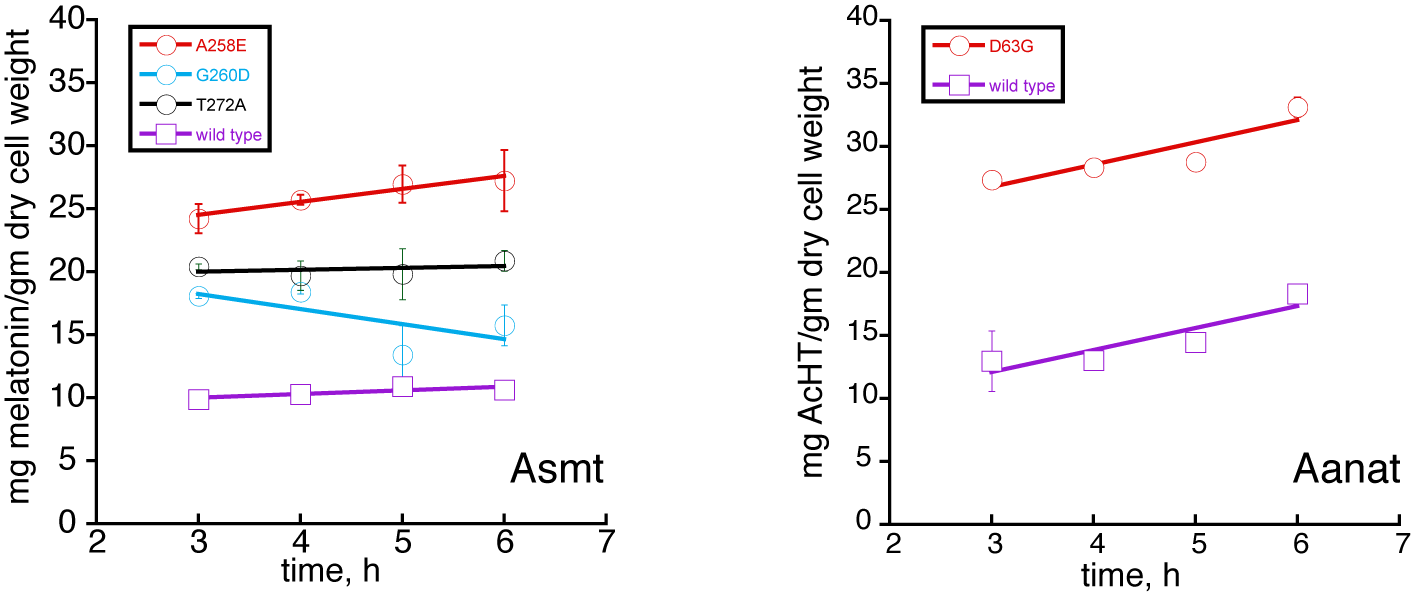

Supplement: S2 Fig — Underlying data can be found in S1 Data. Aanat, aralkylamine N-acetyltransferase; Asmt, acetylserotonine O-methyltransferase. (TIF) [file pbio.2007050.s002.tif]
