# Supplementary material for: Coupling S-adenosylmethionine–dependent methylation to growth: Design and uses
Source: PLoS Biol. 2019 Mar 11;17(3):e2007050. doi: 10.1371/journal.pbio.2007050 (PMC6411097; doi:10.1371/journal.pbio.2007050)
Supplement: S1 Table — Comt, catechol O-methyltransferase. (DOCX) [file pbio.2007050.s003.docx]

| S1 Table: Calculated Z-prime values for evolved Comt isolates at various time of growth | | | |
| --- | --- | --- | --- |
| time, h | HL1817 | HL1818 | HL1819 |
| 2 | 0.65 | 0.79 | 0.67 |
| 3 | 0.90 | 0.89 | 0.87 |
| 4 | 0.88 | 0.94 | 0.93 |
| 5 | 0.96 | 0.97 | 0.92 |
| 6 | 0.96 | 0.97 | 0.94 |
| All strains are Comt ALE isolates derived from ECAH7.  HL1817 carries rpoC (E1146A)  HL1818 carries rpoC (A328P)  HL1819 carries rpoC (HL1819) | | | |
