# Supplementary material for: Coupling S-adenosylmethionine–dependent methylation to growth: Design and uses
Source: PLoS Biol. 2019 Mar 11;17(3):e2007050. doi: 10.1371/journal.pbio.2007050 (PMC6411097; doi:10.1371/journal.pbio.2007050)
Supplement: S2 Table — (DOCX) [file pbio.2007050.s004.docx]

| S2 Table: List of strains used in this study | | |
| --- | --- | --- |
| Strains | Genotype | Comment |
| *E. coli* | | |
| BW25113 | F-, λ-, Δ(araBAD)567, ΔlacZ4787(::rrnB3), Δ(rhaBAD)568, rph-1, hsdR514 | Wild type  [Baba et al. (2006)] |
| ECAH2 | BW25113 ΔcysE | Derived from JW3582 |
| ECAH3 | ECAH2 +pHM11 | Cys3-Cys4 validation |
| ECAH6 | ECAH3 + pMT3 | Pnmt ALE |
| ECAH7 | ECAH3 + pMT7 | Comt ALE |
| HL1815 | BW25113 + pMT3 | Pnmt activity measurements |
| HL1816 | BW25113 + pMT28 | Pnmt activity measurements |
| HL1818 | Evolved isolate of ECAH7 | Growth inhibition assay |
| HMP174 | FolE (T198I) YnbB (V197A) ΔtnaA ΔcysE | Lab stock; BW25113 derived |
| HMP221 | HMP174 ΔmetE ΔmetH | This study |
| HMP229 | FolE (T198I) YnbB (V197A) ΔtnaA ΔtrpR | Lab stock; BW25113 derived |
| HMP231 | HMP229 + pHM6 pHM5 | Asmt activity measurements |
| HMP236 | HMP221 + pHM11 pHM12 | Asmt ALE |
| HMP258 | FolE (T198I) YnbB (V197A) ObgE (E350A) ΔtnaA ΔtrpR (Ptrc::ddc) | Lab stock; BW25113 derived |
| HMP416 | HMP229 + pHM64 pHM5 | Asmt activity measurements |
| HMP417 | HMP229 + pHM65 pHM5 | Asmt activity measurements |
| HMP418 | HMP229 + pHM66 pHM5 | Asmt activity measurements |
| HMP553 | HMP174 Δcfa Tn7-Ptrc::ddc-Ptrc::aanat-Tn7 | Tn7 integration |
| HMP579 | HMP553 + pHM70 pHM79 | Aanat ALE |
| HMP850 | HMP258 ΔyddG (Ptrc::aanat) | Aanat activity measurements |
| HMP851 | HMP258 ΔyddG (Ptrc::aanat (D63G)) | Aanat activity measurements |
| *S. cerevisiae* | | |
| CEN.PK102-5B | *MAT*a *ura3-52 his3Δ1 leu2-3/112* MAL2-8c SUC2 | Background strain [Peter Kötter, Germany] |
| SCAH124 | *MATa ura3-52 his3Δ1 leu2-3/112 MAL2-8c SUC2 met17Δ cho2Δ opi3Δ met2Δ* | This study |
| SCAH134 | SCAH124 + PL_01_D2 | Demonstration in yeast |
| SCAH138 | SCAH124 + pRS415U | Demonstration in yeast |
| Baba, T. et al. *Mol. Syst. Biol.* **2**, 2006.2008 (2006). | | |
