# Supplementary material for: Coupling S-adenosylmethionine–dependent methylation to growth: Design and uses
Source: PLoS Biol. 2019 Mar 11;17(3):e2007050. doi: 10.1371/journal.pbio.2007050 (PMC6411097; doi:10.1371/journal.pbio.2007050)
Supplement: S3 Table — (DOCX) [file pbio.2007050.s005.docx]

| S3 Table: List of plasmids used in this study | | |
| --- | --- | --- |
| Plasmids | Relevant characteristics | Source |
| pBAD33 | Para::MCS Cm^R^ P15A | ATCC |
| pKD46 | Para::gam-bet-exo Am^R^ SC101(ts) | CGSC |
| pCP20 | flp cI857(ts) Am^R^ Cm^R^ SC101(ts) | CGSC |
| pCDF-1b | lacI PT7::lacO::His6::MCS Sm^R^ CDF | Novagen |
| pGRG25 | araC Para::*tnsABCD* attTn7 Am^R^ SC101(ts) | Addgene |
| pHM5 | Ptrc::*ddc* Ptrc::*aanat* Sm^R^ CDF | This study |
| pHM6 | Ptrc::*trpH** ParoF::RBS(recA)::*pcd* Ptrc::*asmt* Cm^R^ P15A | This study |
| pHM11 | PJ23101::RBS(MCD2)::*cys3*::RBS(BB0034)::*cys4* Km^R^ SC101 | This study  [Mutalik, V.K. et al. (2013)] |
| pHM12 | Ptrc::*ddc* Ptrc::*aanat* Ptrc::*asmt* Cm^R^ P15A | This study |
| pHM64 | Ptrc::*trpH(mut)* ParoF::RBS(recA)::*pcd* Ptrc::*asmt (A258E)* Cm^R^ P15A | This study |
| pHM65 | Ptrc::*trpH(mut)* ParoF::RBS(recA)::*pcd* Ptrc::*asmt (G260D)* Cm^R^ P15A | This study |
| pHM66 | Ptrc::*trpH(mut))* ParoF::RBS(recA)::*pcd* Ptrc::*asmt (T272A)* Cm^R^ P15A | This study |
| pHM67 | araC Para::*tnsABCD* Tn7-Ptrc::*ddc* Ptrc::*aanat*-Tn7 Am^R^ SC101(tm) | This study |
| pHM70 | PJ23101::RBS(MCD2)::*cys3*::RBS(BB0034)::*cys4* Ptrc::*asmt (A258E)* Km^R^ SC101 | This study |
| pHM79 | Ptrc::*trpH(mut)* ParoF::RBS(recA)::*pcd* sacB Cm^R^ P15A | This study |
| pMT3 | Pp2::RBS(BCD2)::*pnmt* Cm^R^ P15A | This study  [Mutalik, V.K. et al. (2013)] |
| pMT28 | Pp2::RBS(BCD2)::*pnmt (F214L)* Cm^R^ P15A | This study |
| pMT7 | Pp2::RBS(BCD2)::*comt* Cm^R^ P15A | This study  [Mutalik, V.K. et al. (2013)] |
| PL_01_A9 | CEN/ARS4 *ampR HIS3* P_TEF1_-*SpCAS9*-T_CYC1_ | Jie Zhang (Techenical University of Denmark)  [DiCarlo JE. et al. (2013)] |
| pRS415U | CEN/ARS4 *ampR* pRS415-LEU2 T_ADH1_<-USER cassette->T_CYC1_ | [Jensen ED. et al. (2017)] |
| PL_01_A2 | 2μ ori *ampR* pESC-URA gRNA.*CHO2* | This study  [DiCarlo JE. et al. (2013)] |
| PL_01_A3 | 2μ ori *ampR* pESC-URA gRNA.*MET17* | This study  [DiCarlo JE. et al. (2013)] |
| PL_01_C8 | 2μ ori *ampR* pESC-URA gRNA.*OPI3* | This study  [DiCarlo JE. et al. (2013)] |
| PL_01_E1 | 2μ ori *ampR* pESC-URA gRNA.*MET2* | This study  [DiCarlo JE. et al. (2013)] |
| PL_01_D2 | CEN/ARS4 *ampR* pRS415-LEU2 P*_TDH3_* *_AAAACA_-CaCCS1*-T*_CYC1_* | This study  [Jensen ED. et al. (2017)] |
| Mutalik, V.K. et al. *Nat. Methods* **10**, 354-360 (2013).  Jensen ED, Ferreira R, Jakočiūnas T, Arsovska D, Zhang J, Ding L et al. Transcriptional reprogramming in yeast using dCas9 and combinatorial gRNA strategies. Microb Cell Fact. 2017;16:46.  DiCarlo JE, Norville JE, Mali P, Rios X, Aach J, Church GM. Genome engineering in Saccharomyces cerevisiae using CRISPR-Cas systems. Nucleic Acids Res. 2013 Apr;41(7):4336-43  The following genes were codon optimized for *E. coli* expression: Ddc (Q1IPN9), Aanat (W8QGX9), Asmt (P46597), Pnmt (P10937) and Comt (P21964-2). The gene *CCS1* (Q8H0D3) were codon optimized for *S. cerevisiae* expression. | | |
